# The role of entrepreneurial education in determining actual entrepreneurial behavior: Does TESOL amplified communication apprehension matter?

**DOI:** 10.3389/fpsyg.2022.1074774

**Published:** 2023-01-26

**Authors:** Jianwen Shen, Xuebin Huang

**Affiliations:** ^1^School of Foreign Languages, Guangzhou City University of Technology, Guangzhou, China; ^2^Guangdong Huacheng Industrial and Commercial Technical School, Guangdong, China

**Keywords:** entrepreneurial education, entrepreneurial behavior, TESOL, communication apprehension, psychological capital

## Abstract

Since the impression of innovation is at the fundamental of commercial standing, therefore, Industries estimate innovation as their competitive advantage. Resultantly, industries devote a lot of resources understanding the versatile and dynamic nature of innovations and also keep on progressing innovation techniques and strategies. To meet the objectives of the study, we collected data from the students studying in Chinese universities. A survey questionnaire was designed to collect data from university students who were part of the TESOL program. A total of 224 valid questionnaires were used to proceed with the analysis, where descriptive statistics were calculated using SPSS 21, while hypothesis testing was carried out using Mplus 8. Results revealed the facts that both formal and informal education significantly predicted entrepreneurial behavior, while entrepreneurial intention was also found to mediate the relationship between formal and informal education and entrepreneurial behavior. Similarly, as per prediction, TESOL amplified communication apprehension also significantly moderated the relationships of formal and informal education with entrepreneurial behavior.

## 1. Introduction

Entrepreneurship is one of the key drivers of innovation and it plays a pivotal role in the development of any economy (Woronkowicz, 2021). Furthermore, innovation is referred to as a process of improving and developing ideas to enable them to produce better products and efficient services (Dziallas and Blind, 2019). Since the idea of innovation is at the core of commercial standing, therefore, Industries deem innovation as their competitive advantage. Resultantly, industries spend a lot of resources understanding the dynamic nature of innovations and also keep on improving innovation strategies (Rajapathirana and Hui, 2018).

Entrepreneurial skills taught at university campuses later prove tremendously important in the development of a particular economy. Empirical research suggests that entrepreneurial skills can be honed through the process of education. Furthermore, awareness regarding entrepreneurship as a stable career path can also be raised through education (Ratten and Usmanij, 2021). Given the importance of entrepreneurship, policymakers have tried to provide relevant systems that encourage teaching, learning, and implementing entrepreneurial skills to enhance economic activity. One prime example regarding policy preference can be observed in how the university campuses have been transformed into encouraging students to learn entrepreneurial skills (Portuguez Castro and Gómez Zermeño, 2021). Although empirical research has suggested to a considerable degree that entrepreneurship is a key source of economic development, furthermore, universities have also been modified and improved to accommodate this fact; nevertheless, it remains to be studied how effective this policy shift has been in improving entrepreneurial understandings of the students at university campuses and enabling them to practically implement their entrepreneurial skills to earn a living (Handiman et al., 2022; Usman et al., 2022).

An individual’s entrepreneurism is thought to be inspired by entrepreneurial schooling, which also influences how they view and are passionate about the field (Chuah et al., 2015). According to a different study, training and education in entrepreneurship can influence people’s attitudes and behavioral intentions toward it as well as enhance their managerial skills (Kisubi and Korir, 2021). The goal of entrepreneurial education (EE) is to assist individuals in acquiring entrepreneurial capability, a collection of information, attitude, and a variety of skills (Ndofirepi, 2020). Since it was first conceived, entrepreneurship education has advanced significantly. Recent research supports the notion that entrepreneurship education actively encourages entrepreneurial intention (EI) and enhances entrepreneurial ability (Vodă et al., 2019). According to some academics, entrepreneurship education is the primary factor influencing the improvement of the development of entrepreneurial ability. They also contend that entrepreneurship education can have an impact on and enhance entrepreneurial competence (Stenholm et al., 2021). Few studies, however, take into account how entrepreneurial education influences both entrepreneurial intention and entrepreneurial behavior. Although some academics have hypothesized that business plan contests and entrepreneurial practice projects can boost entrepreneurial ability, enhance entrepreneurial perception, and increase entrepreneurial willingness, this hypothesis is only supported by theoretical construction and has not been empirically supported (Shinnar et al., 2012). Entrepreneurship education has been acknowledged by public policymakers and government organizations all over the world as a way to promote social innovation (Greene and Cooper, 2016). Students receive a foundation of knowledge and are inspired to think entrepreneurially through entrepreneurship education (Kickul et al., 2018). As highlighted in this research, the economy of a nation is built on its entrepreneurial community. Studies do support the belief that a robust economy and sustainable economic setups can be formed by equipping the younger generation (Cardella et al., 2021).

Education in entrepreneurship shapes entrepreneurial behavior and has been a critical driving force behind the expansion of the entrepreneurial sector over the past decade (Bae et al., 2014). Entrepreneurial education is capable of enhancing the appropriate psychological disposition including the inclusion of subjective norms that have an effect on entrepreneurial behavior (Ndofirepi et al., 2022). Individual characteristics, family engagement, and entrepreneurial education may all have a good and significant impact on students’ desire to become entrepreneurs. According to the findings of students who have participated in an entrepreneurship education program have a greater desire of going into business for themselves when they graduate. According to Ismail (2017), there is a higher positive association between education in entrepreneurship and a person’s sense of their own self-efficacy.

Higher entrepreneurial inclinations are demonstrated by students who get an entrepreneurship education. More and more nations are realizing the value of entrepreneurial education as society develops (Unceta et al., 2021). How education might boost entrepreneurship by promoting innovation is a topic of great attention (Shu et al., 2020). To encourage college students to launch firms, the Chinese government strongly promotes entrepreneurial policies and supports “mass entrepreneurship and innovation.” Universities have progressively developed entrepreneurship education programs as a response to national policies, which are essential in encouraging students’ self-employment that strengthens entrepreneurial skills, and heightens entrepreneurial intention. From 2014 to 2016, the Pan-Yangtze River Delta has a greater rate of college graduates launching enterprises 6 months after graduation than other regions (Chien-Chi et al., 2020). Additionally, there is some research value in the fact that college students launch firms with greater zeal. China seeks to establish an atmosphere that is conducive to employment and entrepreneurship, to implement specific plans and programs to assist important groups including recent graduates, migrant workers, and ex-servicemen in finding employment and launching their own firms (Yan et al., 2018).

One of the key abilities graduates may focus on developing is communication, especially if they hope to become entrepreneurs (Avdeeva et al., 2019). The majority of businesses are worried about how well their employees can communicate in groups, organize meetings, interact with others, and give speeches in front of groups (Swaramarinda, 2018). Having weak communication skills will make it difficult for them to find employment (Spinuzzi et al., 2016).

Foreign language apprehension, which is defined as “the sense of tension and concern primarily connected with second language situations, including speaking, listening, and learning,” includes communication anxiety in second languages as one of its subtypes (Tandang and Arif, 2019). Three elements of foreign language communication apprehension were identified by a study, including communication worry, test anxiety, and the fear of receiving a poor grade (Zheng, 2008). A form of shyness known as “communication apprehension” is characterized by fear or uneasiness when speaking to others (Agrawal and Krishna, 2021). A study indicated that many students, even those who experience little stress in other areas of language learning, find speaking in public in the target language to be highly anxiety-inducing (Shi et al., 2015).

A quantitative study that included first-year undergraduate students (non-English majors) examined the relationship between Chinese students’ unwillingness to communicate and their anxiety about learning a foreign language (Foo et al., 2015). The results of the data analysis revealed a significant positive correlation between the two variables. Another study about undergraduates majoring in English examined the Chinese students in both Chinese and English. According to the findings, communication apprehension was adversely connected with both languages, although it was more strongly correlated with students’ readiness to speak in English than Chinese (Peng and Ali, 2015). Keeping in view the findings of such studies on communication apprehension, it is found that there is still a research gap that requires studies on TESOL (Teaching English to Speakers of Other Languages) apprehension and entrepreneurial education and entrepreneurial intention. This study gap in literature became a motivation for the current study which focuses on exploring whether TESOL communication apprehension matters in determining the actual entrepreneurial behavior during entrepreneurial education.

The objective of this study is to establish a framework to study the roles and relationships of the above-mentioned variables simultaneously. The study aims to explore answers to the following research questions: What is the impact of formal and informal education on entrepreneurial behavior? How entrepreneurial intention affects the association between entrepreneurial education and entrepreneurial behavior? What role is played by TESOL amplified communication apprehension, between entrepreneurial education and entrepreneurial behavior? Based on the theory of planned behavior and reasoned actions and after an extensive review of available relevant literature, it was found that no study has been done until now to investigate these research questions. Thus, from the available literature, the current study firstly assumes that formal education and informal education have positive impact on entrepreneurial behavior. Secondly, it is assumed that entrepreneurial intention mediates the positive association between formal and informal education and entrepreneurial behavior. Thirdly, the study assumes the moderating role of TESOL communication apprehension between entrepreneurial education and entrepreneurial behavior.

The study contributes to the research in the following potential ways: First, the study provides an extensive, comprehensive, and systematic overview of the concepts of entrepreneurial education, entrepreneurial behavior, and TESOL communication apprehension. Secondly, this research contributes through its innovative and unique framework of the study, which proposes, and analyzes the relationships and moderator and mediator roles of the variables of this study. Thirdly, the study contributes by adding TESOL communication apprehension into the integrated analytical framework that studies the relationship between green entrepreneurial education, entrepreneurial intention, and entrepreneurial behavior. The framework of this study explains the theoretical perspective in an innovative way. The study also has theoretical and practical implications. Theoretically, it extends the literature on theory of reasoned action and theory of planned behavior while practically it offers guidelines for the stakeholders, educationists, and policymakers. There are five contents included in this study. First, there is a detailed introduction to the study. In the second section, literature review was done and hypotheses were proposed based on the theory of reasoned action and theory of planned behavior. In the third section, the proposed set of hypotheses were tested by data analysis. The fourth section of the study includes the results and discussion and limitations along with theoretical and practical implications of the study.

Therefore, the current investigation provides insights into the concepts of entrepreneurial education and entrepreneurial behavior. Moreover, this study also hypothesized that Entrepreneurial Education (EE) and Entrepreneurial Behavior (EB). High risks and uncertainties are always involved in an entrepreneurial venture, thus communication apprehension increases these risks manifolds. Based on the theory of planned behavior, it is explored in this study that working on TESOL communication apprehension can bring positive outcomes for students and educationists. This study collected the data from 224 TESOL course students from various universities in China under a convenient sampling technique. The present study applied PLS-SEM for empirical analyses using Smart PLS software. Additionally, by conducting Simp-Slope analysis, researchers have further confirmed the moderating effect of CA at different low, medium, and high corresponding values. It was found that higher values of CA moderated the relationship more strongly and positively as compared to medium and lower values. Moreover, TESOL amplified communication apprehension also significantly moderated the relationships between formal and informal education with entrepreneurial behavior.

## 2. Literature review and hypothesis development

In the process of developing ideas, models, and theories, the theory of reasoned action came to the fore as it was able to predict attitudes and behaviors (Mi et al., 2018). However, later on, deficiencies were also found in this theory and it was enhanced into the theory of planned behavior (Ajzen, 2020). This theory is different as it incorporates perceived behavioral control. The theory suggests that behavior can be predicted by attitude toward subjective norms and perceived behavioral control. Therefore, drawing a complete picture in the light of this theory, intentions shape a behavior, attitude toward a particular behavior refers to motivational factors that influence the behavior, subjective norms exert pressure embedded in an individual’s surroundings that work as an enforcer, and the perceived behavioral control is the ability of the individual to exhibit a particular behavior (Ajzen, 2020; Sumiati et al., 2021).

As this theory has universal applicability Vis-à-vis behaviors, therefore, it is also relevant to be used in the case of entrepreneurial behaviors since these behaviors are the outcome of cognitive planning and not merely reactions to an outside stimulus. This is how a new model for entrepreneurial behavior (EB) can be carved up that can explain attitudes toward entrepreneurial ventures, subjective norms, and perceived entrepreneurial abilities (Abbasianchavari and Moritz, 2021). This model has frequently been used to predict entrepreneurial tendencies in individuals as demographic characteristics alone have proven to be significantly inefficient in reaching a reliable conclusion in this matter. This also explains why the theory of planned behavior has frequently and successfully been used to investigate the relationship between entrepreneurial education and entrepreneurial behavior.

It has been argued that entrepreneurial education has the potential to lead to the adoption or formulation of entrepreneurial behavior. Furthermore, entrepreneurship lays a stable foundation for the erection of sustainable economic growth, thus, it certainly adds to economic development. A recent trend in research regarding entrepreneurship has shifted its focus from trait theory to the perspectives of cognition in entrepreneurship (Su et al., 2020). One of the key indicators of entrepreneurial theory is the term “entrepreneurial performance.” Entrepreneurial performance refers to the achievements and efficiency of the new generations of entrepreneurs (Bian et al., 2020).

### 2.1. Entrepreneurial education (EE) and entrepreneurial behavior (EB)

The process of education involves teaching as well as learning. There can be many different forms that can be described as learning. However, formal learning can be described as a process of structured learning that does not occur in a working environment and that typically takes place in a classroom in formal educational settings (De Troyer et al., 2020). Formal learning is also termed as a process that takes place in a standard paradigm of learning. There are standard pedagogical frameworks with didactic interaction to govern the process of formal learning (Viberg et al., 2021). However, the process of formal learning in a workplace is embedded in the idea to inculcate new information of skills in the employees in order to improve their knowledge base or skill set so that they be enabled to perform their respective duties more efficiently. Furthermore, two other important components of formal learning are institutional sponsorship and sponsored learning modules. Therefore, formal learning or training at work is also carried out in a specific context that enables the learners to sharpen a particular aspect of their skill set or get accustomed to an entirely new skill or set of knowledge in order to become efficient at their jobs. The formal learning process consists of a specific learning framework, a particular learning event, availability of designated teachers, award of a certificate or credit, and specific outcomes (Spaan et al., 2016).

Whereas, on the other hand, informal learning can be termed as a process of learning that is “not formal” (Rogers, 2014). This definition rightly seems simplistic though, however, it is for a reason that there actually are problems with the definition. The term has been used with many different contours in different texts. A large number of texts simply assume the definitions or use these terms without much attention to the context of them. A small portion of the research attempts to define these loosely understood terms; however, their volume is too small to be considered something considerable. There is not overwhelming agreement regarding how these terms should be defined in the literature and this fact encourages the use of assumptions in the use of such terms. Different definitions and understanding do overlap each other, however, a general consensus is absent. For instance, one method of referring to informal learning suggests that informal learning can take place even in the absence of an organized learning program at a workplace. It goes on to stress that knowledge and skill at a workplace can be gained without attending or being part of learning programs that are sponsored by the institutions.

And this is the very characteristic that makes the process of informal learning “informal” in the first place. The process of learning is a fluid process that occurs even in moments of the need for a particular requirement of skill exhibition. Learning can occur even at times when the intention of learning is absent, particularly in a work setting. This definitional context highlights the fact that how the process of informal learning depends on factors such as individual curiosity, self-efficacy, and cognitive orientation (Cox, 2018).

Entrepreneurial intent is vital (Usman et al., 2022). The urge to establish a new firm, also known as entrepreneurial ambition, is an alternative career path to traditional employment. According to many researchers, intentions are the most significant indication of entrepreneurial action. According to previous research, entrepreneurial goals have a favorable and substantial effect on entrepreneurial activity (Lucas et al., 2016). Over the last three decades, a significant body of research has explored the impact of entrepreneurial zeal on projecting entrepreneurial intention and direction. Much research has not been done on how a person’s level of ardor for an enterprise affects how an enterprise develops.

There has not been enough research on what elements determine whether or not an individual takes an entrepreneurial risk (enthusiasm, awareness, self-efficacy, and proactive character). We built a quantitative link between goals and activities to bridge this gap and advance the state of entrepreneurship research (Cardon et al., 2009). We discovered that aspirations to be an entrepreneur did not always lead to actual entrepreneurship when we tested a conceptual model of the intention-behavior gap in this subject. When the intention and behavior model is evaluated, researchers discovered that entrepreneurial goals could only partially explain the variability in entrepreneurial activity (Cardon et al., 2009). Therefore, it is not compulsory for informal learning to occur if the intention to learn is non-existent. Everyday working experience that involves working toward achieving a certain goal or problem solving may have instances of informal learning.

Furthermore, there may also not be a formal coach or teacher but rather an individual may be consulted in a bid to solve some problem or learn about a solution, considering that individual is better equipped or expert in a particular field area. Furthermore, informal learning may also not follow a strict and predictable learning pattern that is required to be followed in the case of formal learning.

Informal learning occurs whenever there is a need to learn a particular aspect of something (Wu et al., 2021). It occurs only when there is a specific requirement for learning about something. The development of intercultural awareness necessitates an openness to being present in various multicultural setting. Respect for another culture may be shown by cultivating an awareness of how our worldview is influenced by cultural context and by allowing for the expression of other points of view.

Entrepreneurial behavior is believed to have a lot to do with our formality and informality. Formal education can strengthen these tendencies while informal education can change them or make them weaker. Entrepreneurial behavior is shaped by two types of education: formal and informal. According to Charles Elson, formal education expands one’s knowledge of basic skills through reading, writing, and math. Informal educations, on the other hand, focus on critical thinking, creativity, and problem solving. While both are meant to enhance entrepreneurial skills, conversing within entrepreneurship reveals that formal educations have a significant impact due to their role in guiding students through a process of self-study and providing them with a sense of mastery over particular topics or skill sets; without it. Entrepreneurial behavior can be enhanced through positive learning experiences and experiences in the classroom. This is achieved by having an instructor in the class who has a positive attitude toward entrepreneurship and who inspires students to seek out opportunities. The instructor should also be creative, versatile, and spontaneous so that their new ideas can challenge students’ thought processes.

Keeping this in view, a study attempts to define informal learning as an institutional process that the acquisition of a particular sort of knowledge, a specific set of skills, values, and even feelings at its core only to enhance the focus and productivity of individuals and teams (Rudeloff, 2019). Furthermore, some sanctioned learning may also be referred to as informal learning such as coaching, mentoring, or special assignments. There are four broad conceptualizing principles of informal learning (Watkins and Marsick, 2021). These four principles are as follows: context (learning that occurs outside of a formal educational setting), cognizance (having the intention to learn), experiential (learning that involves practice and judgment), and relationship (learning that involves coaching and teamwork). The above-mentioned definitions and conceptualizing characterizations contend that informal learning is highly important and effective. Furthermore, it is also significant to underline that informal learning is not an ‘inferior’ form of learning in any way if compared to the formal learning practices that mostly take place in a classroom environment (Marsick and Watkins, 1990). From the above discussion, it is hypothesized that formal education and informal entrepreneurial education have positive impact on entrepreneurial behavior.

*H1*: Formal education has a positive impact on entrepreneurial behavior.

*H2*: Informal education has positive impact on entrepreneurial behavior.

### 2.2. Entrepreneurial intention as a mediator between EE and EB

According to entrepreneurial intention is considered to be a significant antecedent in the process of driving entrepreneurial action. If there is no purpose to engage in entrepreneurial behavior, then there will be no entrepreneurial activity. Education in entrepreneurship, which has been studied for many years, has been shown to encourage students’ ambitions to start their own businesses. It is also considered a crucial indicator of the effect that entrepreneurship education has had on people’s intentions to start a business. The study that has been done so far reveals that entrepreneurship education motivates college students to gain knowledge and skills relevant to entrepreneurship and changes the students’ ways of thinking. Students can be motivated to gain a more comprehensive understanding of entrepreneurship through entrepreneurship education, which improves their entrepreneurial self-efficacy and opportunity recognition ability. This has the effect of indirectly influencing entrepreneurial intention.

As entrepreneurship is recognized as the outcome of planned behavior, therefore, it might not be possible for an individual to engage with an entrepreneurial opportunity in the absence of entrepreneurial intention (Mahfud et al., 2020). EI is significant since it represents the self-acknowledged belief and conviction of an individual regarding going down that path of entrepreneurship in the future (Anjum et al., 2021). A study noted that EI is positively associated with the establishment of entrepreneurial ventures in a society (Mei et al., 2020). Therefore, it can be contended that the level of EI truly reflects the level of entrepreneurial potential in a particular country (Syed et al., 2020). Hence, if the aim is to promote entrepreneurship in a society, it is of paramount importance that the consequences and antecedents of EI are understood properly (Sansone et al., 2021). If there is a favorable perception with respect to entrepreneurship, the atmosphere will become fertile (Ajzen, 2020). The results of a study suggest that personal attitude and perceived behavioral control were the primary determinants of EI (Newbery et al., 2015).

This study suggests that EI mediates between entrepreneurial education and entrepreneurial behavior. It is contended that an individual having EE is more prone to express his/her EI based on his/her confidence to play certain entrepreneurial roles. The same is also supported by the theory of planned behavior according to which if the perceived behavioral control with regard to entrepreneurship is higher, the intention regarding starting up a business would also be higher (Hwee Nga and Shamuganathan, 2010). Furthermore, there are other studies as well that assert the same that EI significantly influences EB (Kickul et al., 2009). As studies have empirically established that intentions are a solid predictor of behavior, therefore, it is contended here that the presence of strong EI makes the initiation of entrepreneurial ventures highly likely (Mouraviev and Avramenko, 2020). As a matter of fact, there is overwhelming empirical evidence provided by the studies focused on the United Kingdom, Finland, and Austria that well-informed intentions of individuals are most likely to translate into starting an entrepreneurial business set-up (de Meza et al., 2019). The above research findings can be used to assume that entrepreneurial intention mediates the positive association between formal and informal education and entrepreneurial behavior.

Entrepreneurial intention has a significant influence on entrepreneurial behavior. It is related to the extent to which entrepreneurs can adopt the cognitive and emotional components necessary for effective decision-making, and implement effective actions inspired by their motivation this is particularly true in periods of uncertainty and high information overload. Entrepreneurs who rely on motivation alone may not take advantage of new opportunities when they arise, as they fail to take advantage of knowledge gained through business education. Entrepreneurial intention is an essential component of entrepreneurial behavior. Entrepreneurial intention describes an entrepreneur’s motives and intentions regarding the formation, operation, and growth of a new venture. Entrepreneurial intentions are the direct result of behavioral predispositions, and intention is easier to change than behavior.

*H3*: Entrepreneurial intention mediates the positive association between formal education and entrepreneurial behavior.

*H4*: Entrepreneurial intention mediates the positive association between informal education and entrepreneurial behavior.

#### 2.2.1. TESOL amplified communication apprehension as a moderator between EE and EB

Before drawing the relationship between communication apprehension with EE and EB, it is pertinent to define communication apprehension. There is one definition that is widely recognized as the most comprehensive definition of communication apprehension. According to this definition, communication apprehension is an anxiety that is caused by the actual or expected relationship with the individual or other (Soleimani and Bahramnia, 2018). Such apprehension results in the desire to escape. There are plenty of indications on the issues of escape; however, the interest of social scientists and psychologists has grown significantly in the last two decades.

The definition of communication in the entrepreneurial realm contends that it is the level of proficiency in communication that is required by entrepreneurs to communicate with other stakeholders (Avdeeva et al., 2019). Establishing a business requires significant communication skills, particularly at the initial stages of business (Peña-Acuña and Sánchez-Cobarro, 2017; Al-Musalli, 2019; Khamis and Wahi, 2021). A study considers it a social skill and categorizes it into four different aspects (Baron and Armstrong, 2007). The study maintains that this social skill consists of the ability to form accurate perceptions regarding others, the ability to establish a firm and positive first impression, the ability to express emotions accurately, and the ability to act appropriately in accordance with the social situation (Baron and Armstrong, 2007).

However, a study found that the graduates who had clumsy communication skills were not able to either establish strong EI or EB (Buschow and Laugemann, 2020). Apart from having EI or EB, university graduates were found to be lacking social skills such as communication skills to be able to make their way proficiently into the employability market (Lyons and Campbell, 2022). The importance of communication skills remains one of the most studied topics; however, interestingly, there has not been much attention to the fact how students struggle to overcome the deficiencies in their communication skills (Iksan et al., 2012). One of the biggest hurdles in this regard is communication apprehension (CA; Lie, 2018). It is referred to as a type of fear that bars one from communicating with his/her colleagues out of fear and that resultantly affects his/her abilities to perform efficiently in the workplace (Shi et al., 2015).

A high level of CA leads to negative emotional tendencies that deteriorate one’s resolve to perform his/her duties (Cavanaugh, 2015). Higher CA leads to fear of communication with others which makes one appear very quiet as it is one of our natural tendencies to not indulge in a practice that we are fearful of. Therefore, it has been observed that people with higher CA tend to prefer jobs or roles that do not require them to communicate with others on a regular basis as they look to keep their communication with others to the bare minimum level. Contrarily, those who have low CA tend to prefer jobs and roles that provide them with the opportunity the engage with others as they always welcome the prospect of having a wider, prolonged, and consistent channel of communication with others. Nevertheless, graduates with higher CA can still make a good career despite having a prolonged fear of lacking communication skills by establishing their own entrepreneurial ventures. Creating one’s own business and being self-employed works as a catalyst for those whose self-esteem is not that high given their overwhelming fear of communication. The same has been supported by a study that focused on Malaysian graduates who were not proficient in English and still were able to survive in the job market by creating their own alternative opportunities using their EI and EB (Sarudin et al., 2013).

There has been a visible consensus among researchers that effective, strong, and good communication is highly essential for entrepreneurial bids (Spinuzzi et al., 2016). However, this study contends that contrary to popular perception, strong communication skills are the bedrock of EI and EB, and that it is the lack of communication skills that encourages individuals to opt for entrepreneurial options as alternate career paths as they feel more confident and less stressful. This is also supported by many past studies that poor communication skills lead many individuals jobless as they fail to impress employers. However, it also opens an alternative pathway of entrepreneurship that these individuals tend to opt for with ease. From the above-mentioned facts about communication apprehension, it is assumed that TESOL amplified communication apprehension moderates the positive association between formal and informal education and entrepreneurial behavior.

*H5*: TESOL amplified communication apprehension moderates the positive association between formal education and entrepreneurial behavior.

*H6*: TESOL amplified communication apprehension moderates the positive association between informal education and entrepreneurial behavior.

## 3. Methodology

Students in China have been the biggest target audience for TESOL activities (Borg and Liu, 2013). Therefore, we selected the 20 universities operating in Beijing where we randomly selected students who participated in EAP programs and took courses of entrepreneurship. Therefore, for data collection, a validated survey questionnaire was circulated to the selected universities. A total of 360 questionnaires were circulated to the concerned universities and out of which 250 questionnaires were filled, whereas 224 questionnaires were found to be valid (response rate 62%). These validated questionnaires were considered for further analysis.

## 4. Results and discussion

### 4.1. Measurement model

It is mandatory to analyze the measurement model first before proceeding further. Thus, we assessed convergent validity and discriminant validity prior to the assessment of structural model was assessed (Anderson and Gerbing, 1988). For this purpose, premier software in quantitative data analysis “Mplus” was applied. Mplus not only has the capacity to use normal data, but it can also handle the non-normal data (Van Der Linden et al., 2017). Moreover, to determine the “model fit,” we used goodness of fit indices, i.e., Chi-square, root mean square error of approximation (RMSEA), comparative fit index (CFI), Tucker–Lewis index (TLI), and standardized root mean square residual. According to Hu and Bentler (2009), RSMEA is considered excellent if it remains less than 0.05 and is acceptable even if it remains up to 0.08, SRMR is acceptable up to 0.08 and is categorized as excellent if it remains less than 0.05, CFI, TLI > 0.90 are satisfactory and > 0.95 are considered as excellent, and SRMR value is also acceptable if it ranges up to 0.08. Table 1 provides complete details about the model fit indices.

**Table 1:** Model fitness.

Model	*χ*^2^	*χ*^2^/df	CFI	TLI	RMSEA	SRMR
Quality criteria	>0	<5	>0.9	>0.9	≤0.8	≤0.8
Indices	1,211	1211/727 = 1.65	0.942	0.938	0.055	0.048

*χ*^2^, Chi-square value; *df*, degree of freedom; RMSEA, root mean square error of approximation; CFI, comparative fit index; TLI, Tucker–Lewis Index; SRMR, standardized root mean square residual.

According to Hair (2009), convergent validity is assessed based upon the standardized loadings of all the items of the construct/scale. However, for an item of the construct to be considered for determining the convergent validity, its standardized loadings should be greater than 0.5, which is in this case. The main research instrument contained 42 items and the standardized loadings of the items ranged from 0.583 of IFED-3 to EB-2 0.913. These values proved that all the items show strong evidence of convergent validity (see Table 2). Moreover, to ensure the internal consistency of the scale, we have calculated composite reliability (CR) and average variance extracted (AVE) of the constructs. As the study aimed at theory testing, we conducted confirmatory factor analysis. According to the criteria, for a construct to have internal consistency, it is recommended that the values of CR remain at least 0.7 or greater, while the values of AVE should at least remain 0.5 (Fornell and Larcker, 1981). The results in Table 2 revealed that the scale meets the required criteria of reliability.

**Table 2:** Convergent validity and internal consistency reliability.

Latent variable	STDYX	CR	AVE
**Informal Education (EFED) by**		0.87	0.54
IFED1	0.601		
IFED2	0.637		
IFED3	0.583		
IFED4	0.848		
IFED5	0.853		
IFED6	0.812		
**Formal Education (FED) by**		0.92	0.66
FED1	0.769		
FED2	0.857		
FED3	0.866		
FED4	0.827		
FED5	0.816		
FED6	0.720		
FED7	Deleted		
**Entrepreneurial Intention (EI) by**		0.92	0.74
EI1	Deleted		
EI2	0.871		
EI3	0.871		
EI4	0.854		
EI5	0.845		
EI6	0.872		
**Entrepreneurial Behavior (EB)**		0.96	0.78
EB1	0.888		
EB2	0.913		
EB3	0.881		
EB4	0.881		
EB5	0.872		
EB6	0.877		
EB7	0.866		
**TESOL Amplified Communication Apprehension (CA) by**		0.95	0.66
CA1	0.807		
CA2	0.809		
CA3	0.809		
CA4	0.824		
CA5	0.866		
CA6	0.854		
CA7	0.811		
CA8	0.805		
CA9	0.817		
CA10	0.737		
CA11	0.809		
CA12	0.780		
CA13	0.838		
CA14	0.846		
CA15	0.826		
CA16	0.753		

*n* = 224, CR, composite reliability; AVE, average variance extracted; π = standardized loadings.

### 4.2. Analyzing correlation and discriminant validity

Moreover, in order to decide whether the scale has discriminant validity or not, comparing the squared root of the AVE with the correlation coefficient is essential (Fornell and Larcker, 1981). Diagonal bolded values in Table 3 represent the squared root of AVE, while other values indicate the strength of correlation amongst variables of the study. It is evident from Table 3 that diagonal bolded values are considerably greater than the correlation coefficients of the relevant constructs which confirms that the constructs have measured for what they are designed to measure.

**Table 3:** Correlation and discriminant validity.

Variables	FED	IFED	EI	EB	CA
FED	0.731				
IFED	0.511	0.810			
EI	0.292	0.264	0.862		
EB	0.470	0.423	0.605	0.882	
CA	0.230	0.205	0.442	0.398	0.812

All values were significant **p < 0.05, FED, formal education; IFED, informal education; EI, entrepreneurial intention; EB, entrepreneurial behavior; CA, communication apprehension.

### 4.3. Data variability

The variability of the data was assessed by analyzing the mean values and standard deviation from the mean. The mean score of the variables ranged from 5.037 of formal education to 6.42 of TESOL amplified communication apprehension. Whereas, the standard deviation of the constructs ranged from 0.894 of formal education to 1.220 of entrepreneurial behavior. The values of standard deviation are well within the prescribed range and the data are good to enough to be handled using Mplus (Van Der Linden et al., 2017). Table 4 provides more details about data variability.

**Table 4:** Descriptive statistics.

Variables	Mean	Std.
Formal education	5.037	0.993
Informal education	5.070	0.896
Entrepreneurial intention	5.977	0.894
Entrepreneurial behavior	5.387	1.220
TESOL amplified communication apprehension	6.427	0.771

Std., standard deviation of the construct.

### 4.4. Hypothesis testing

Table 5 contains information about the hypotheses of direct relationships. As pointed out by Henseler et al. (2009), the standardized coefficients are the same as the regression coefficients, and that is why the decision about the hypothesis is made based upon the standardized coefficients and their significance (value of *p*) provided in Mplus 7 output.

**Table 5:** Direct relationship.

Hypothesis	Β-value direct	*p*-value	Outcomes
H1: EB ON FED	0.439	0.001	Supported
H2: EB ON IFED	0.496	0.000	Supported

FED, formal education; IFED, informal education; EI, entrepreneurial intention; EB, entrepreneurial behavior.

**Figure 1:**
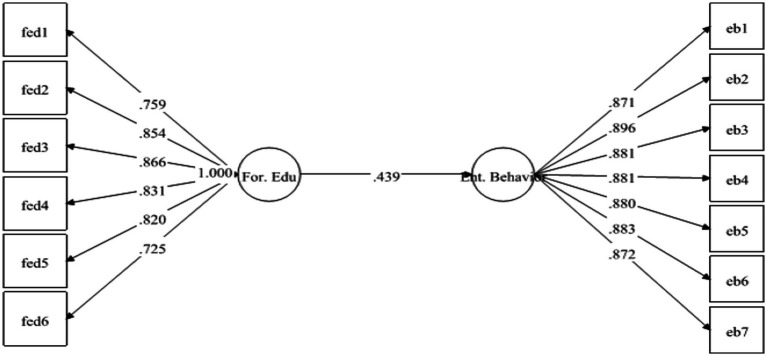
Direct relationship between formal education (For. Edu) and entrepreneurial behavior (Ent. Behavior).

**Figure 2:**
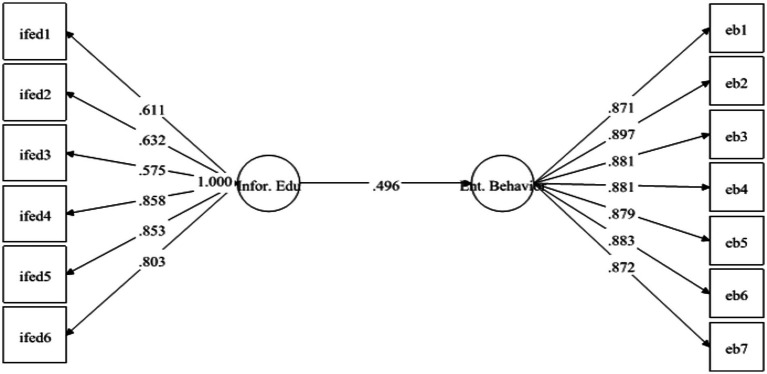
Direct relationship between informal education (Infor. Edu) and entrepreneurial behavior (Ent. Behavior).

The results have demonstrated that EB is strongly positively linked to FED with *β* = 0.439 and is significant at 0.001. Therefore, *H*1 was supported (see Figure 1). Likewise, *H*2 examined the relationship between IFED and EB. The results have exposed that IFED has positively predicted EB with *β* = 0.496 and value of *p* 0.00. Hence *H*2 was also found to be supported (see Figure 2).

Additionally, results in Table 6 have explained the indirect association between IVs and DVs. As per the requirement, mediation analysis was performed to confirm whether the intervening/mediating variable (MV) enhanced the impact of IV to the DV (Hair, 2011).

**Table 6:** Hypothesis testing for mediation.

Hypothesis	*β*-value SIE	95% SIE	Outcomes
H3: Mediation of EI between FED and EB	0.162 (0.00)	0.104—0.221	Supported
H4: Mediation of EI between IFED and EB	0.178 (0.00)	0.120---0.236	Supported

SIE, specific indirect effect; All values are significant **p < 0.05, Stdyx, standardized beta coefficient; CI, confidence interval; FED, formal education; IFED, informal education; EI, entrepreneurial intention; EB, entrepreneurial.

**Figure 3:**
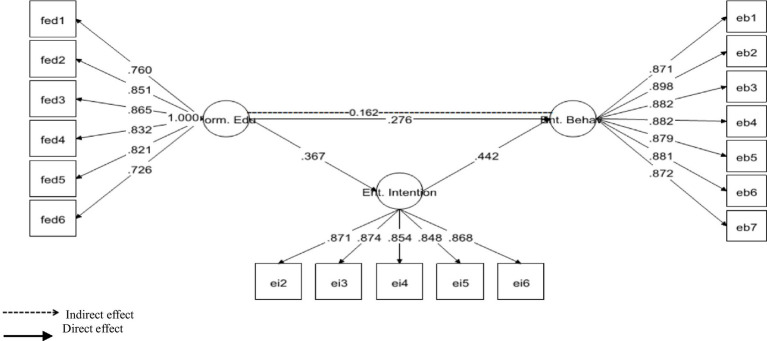
Mediation of entrepreneurial intention (Ent. Intention) between formal education (Form. Edu) and entrepreneurial behavior (Ent. Behavior).

We opted for, “bootstrapping procedures” to test the significance of the mediation path (Preacher and Hayes, 2004; Preacher et al., 2010). Moving ahead with the analysis, it was found that specific indirect effect remained significant as the STDYX of SIE (specific indirect effect) was 0.162 (0.00) with a 95% CI [0.104–0.221]. Because the upper CI and lower CI values do not include zero, therefore, it is confirmed that “EI” positively and significantly mediated the relationship between “FED” and “EB.” Thus *H*4 was supported (see Figure 3).

**Figure 4:**
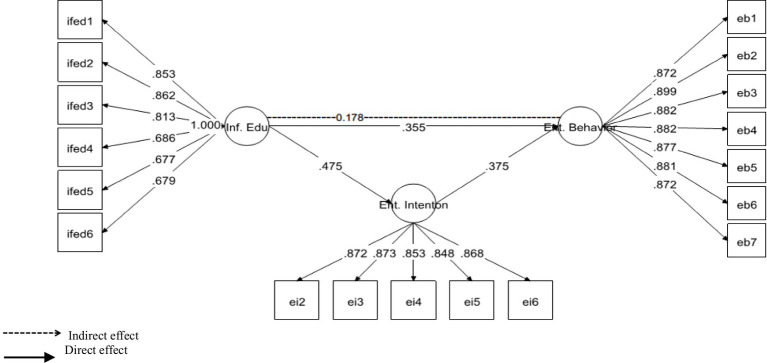
Mediation of entrepreneurial intention (Ent. Intention) between informal education (Inf. Edu) and entrepreneurial behavior (Ent. Behav).

Likewise, in order to investigate the mediating role of EI between Inf. Edu. (Informal Education) and Ent. Behavior (Entrepreneurial Behavior), we proceeded with mediation analysis. Notably, Mplus applies “IND” command to analyze the specific indirect effect of the mediated relationship. Henceforth, it was revealed that EI significantly and positively mediated the relationship of informal education-entrepreneurial behavior with *β* = 0.178, *p* = 0.00 (see Figure 4). Moreover, in order to further confirm the specific indirect effect, we opted for, “bootstrapping procedures” to test the significance of the indirect path (Preacher and Hayes, 2004; Preacher et al., 2010). It was confirmed that the 95% CI did not include zero between the upper and the lower values of CI [0.120–0.236]. This indicates that as per prediction, Hypothesis 4 was supported.

**Figure 5:**
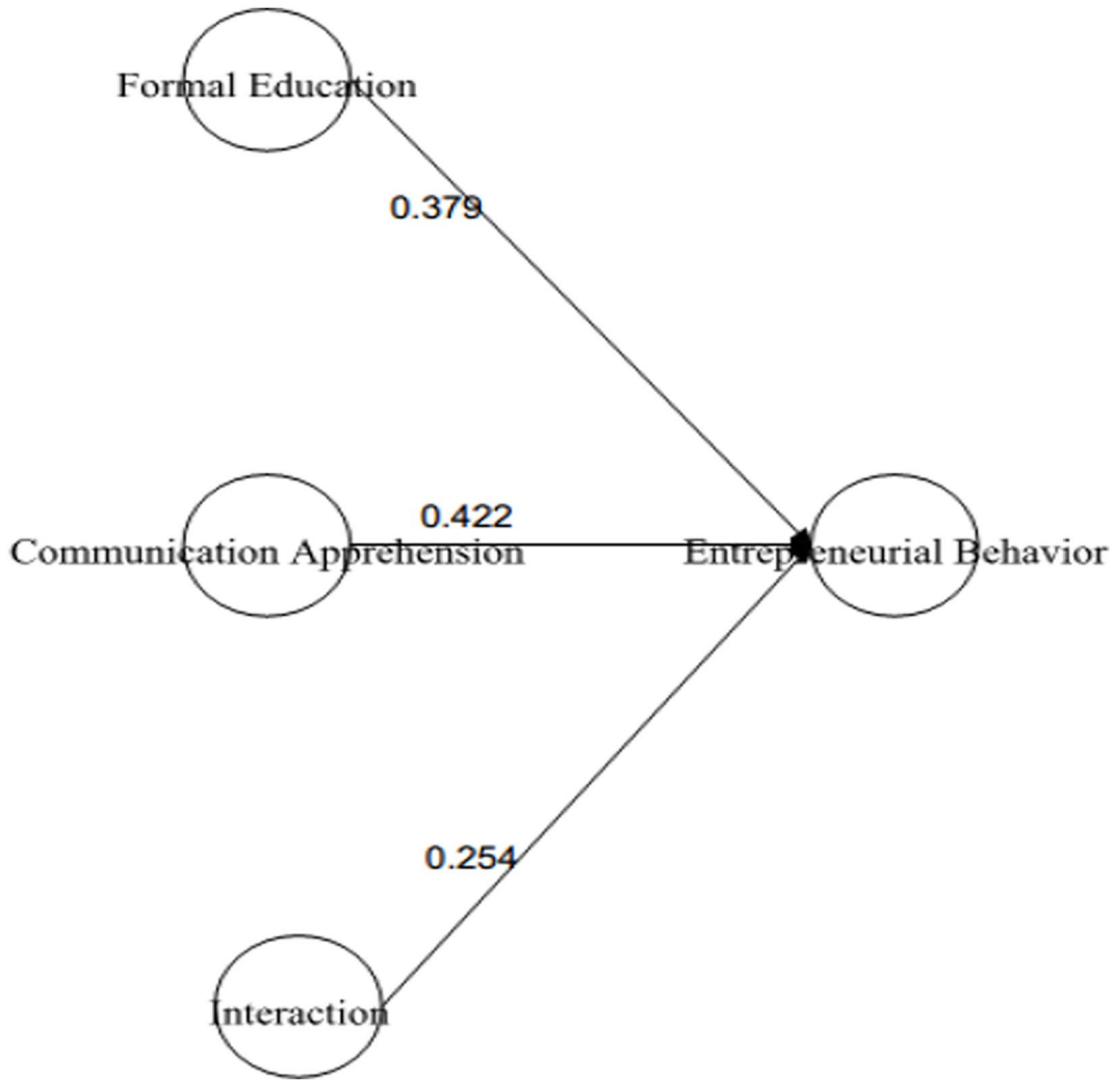
Moderating effect of TESOL amplified communication apprehension on the relationship of formal education—entrepreneurial behavior.

**Figure 6:**
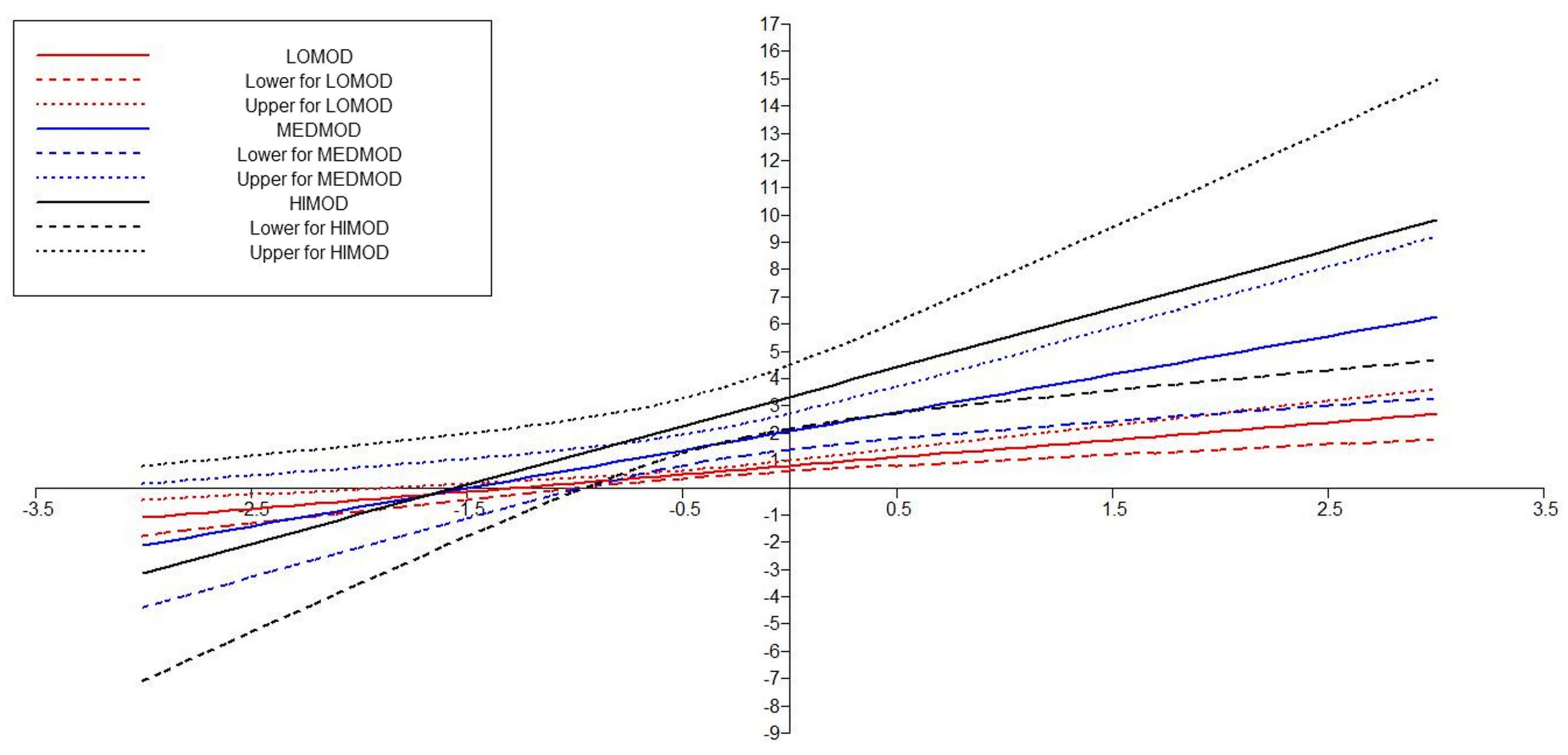
Interaction plot showing the moderating effect TESOL amplified communication apprehension on the relationship of formal education—entrepreneurial behavior.

We also investigate the moderating role of CA on two paths. First, we examined the moderating role of CA on the relationship of FED-EB. To proceed with the analysis, we applied “X with” technique for creating the interaction term between concerned IV-MV (Van Der Linden et al., 2017) and for assessing the effect of interaction term. The results in Table 7 highlighted that the direct association FED--EB was significant *β* = 0.379 (*p* = 0.00) with 95% CI [0.247–0.510], the direct association of the moderating variable CA with EB (CA-EB) was also significant with a *β* = 0.422 (*p* = 0.000) and 95% CI [0.278–566] and the interaction term (CA X FED) also remained significant *β* = 0.254 (*p* = 0.018) with a 95% CI [0.078–0.431]. All values were significant because the corresponding *p*-values are <0.05 and the 95% CI did not include zero. These results have exposed that *H*5 was supported. Researchers have further confirmed the moderating effect at different low, medium, and high values of moderating variables. It was found that higher values of CA moderated the values more strongly and positively as compared to medium and lower values (see Figures 5, 6).

**Table 7:** Hypothesis testing for moderation analysis.

Hypothesis	Direct relationship	Moderation (interaction effect)	95% CI	Outcomes
H5: Moderating role of CA on the relationship between FED and EB	EB ON FED = 0.379 (0.000)		[0.247–0.510]	Supported
	EB ON CA = 0.422 (0.000)		[0.278–566]	
		0.254 (−0.018)	[0.078–0.431]	
H6: Moderating role of CA on the relationship between IFED and EB	EB ON IFED = 0.556 (0.00)		[0.374–0.739]	Supported
	EB ON CA = 0.369 (0.00)		[0.227–0.510]	
		0.245 (0.035)	[0.054–0.436]	

FED, formal education; IFED, informal education; EI, entrepreneurial intention; EB, entrepreneurial behavior; CA, communication apprehension; Stdyx, standardized beta coefficient; CI, confidence interval.

**Figure 7:**
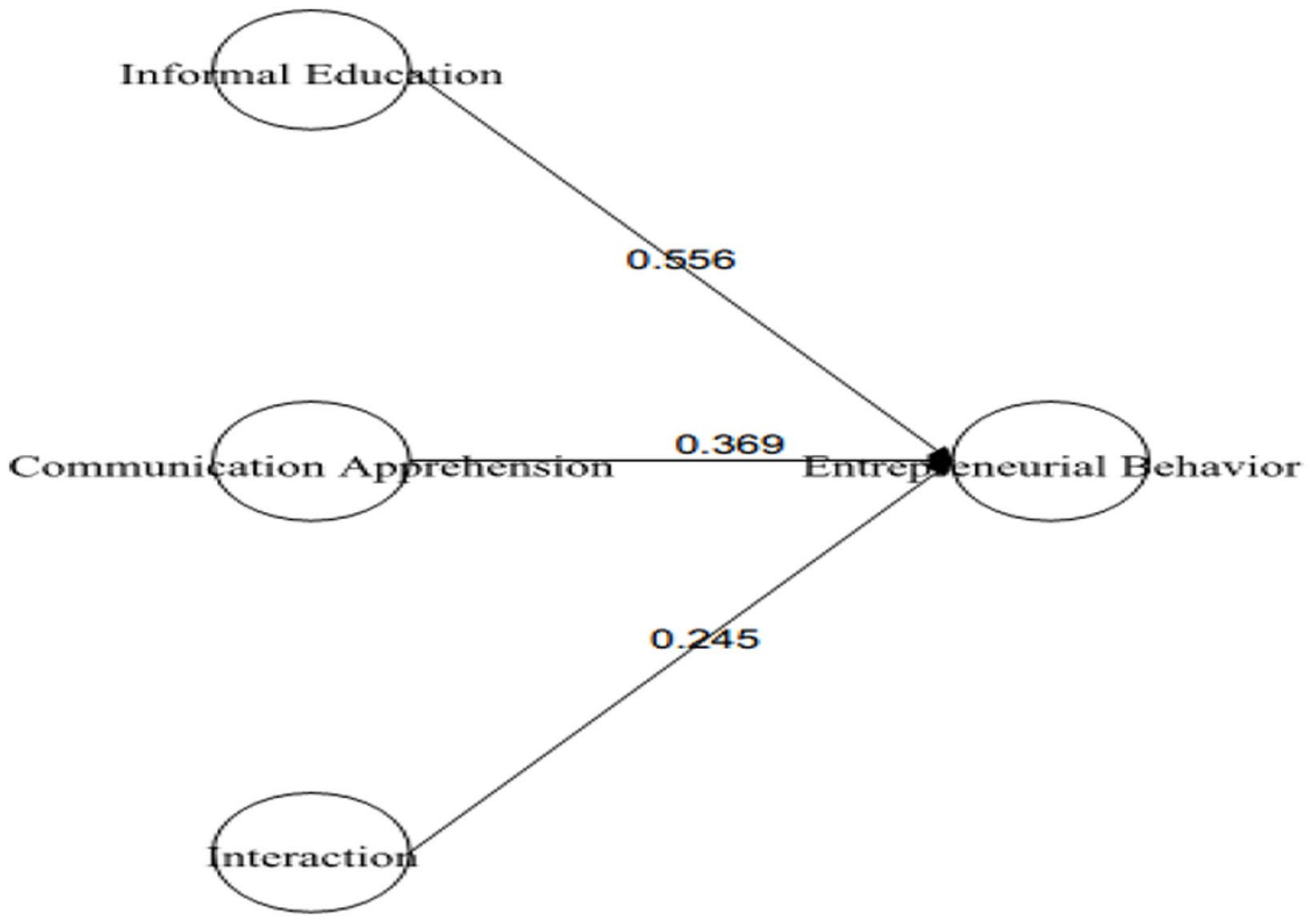
Moderating effect of TESOL amplified communication apprehension on the relationship of informal education—entrepreneurial behavior.

**Figure 8:**
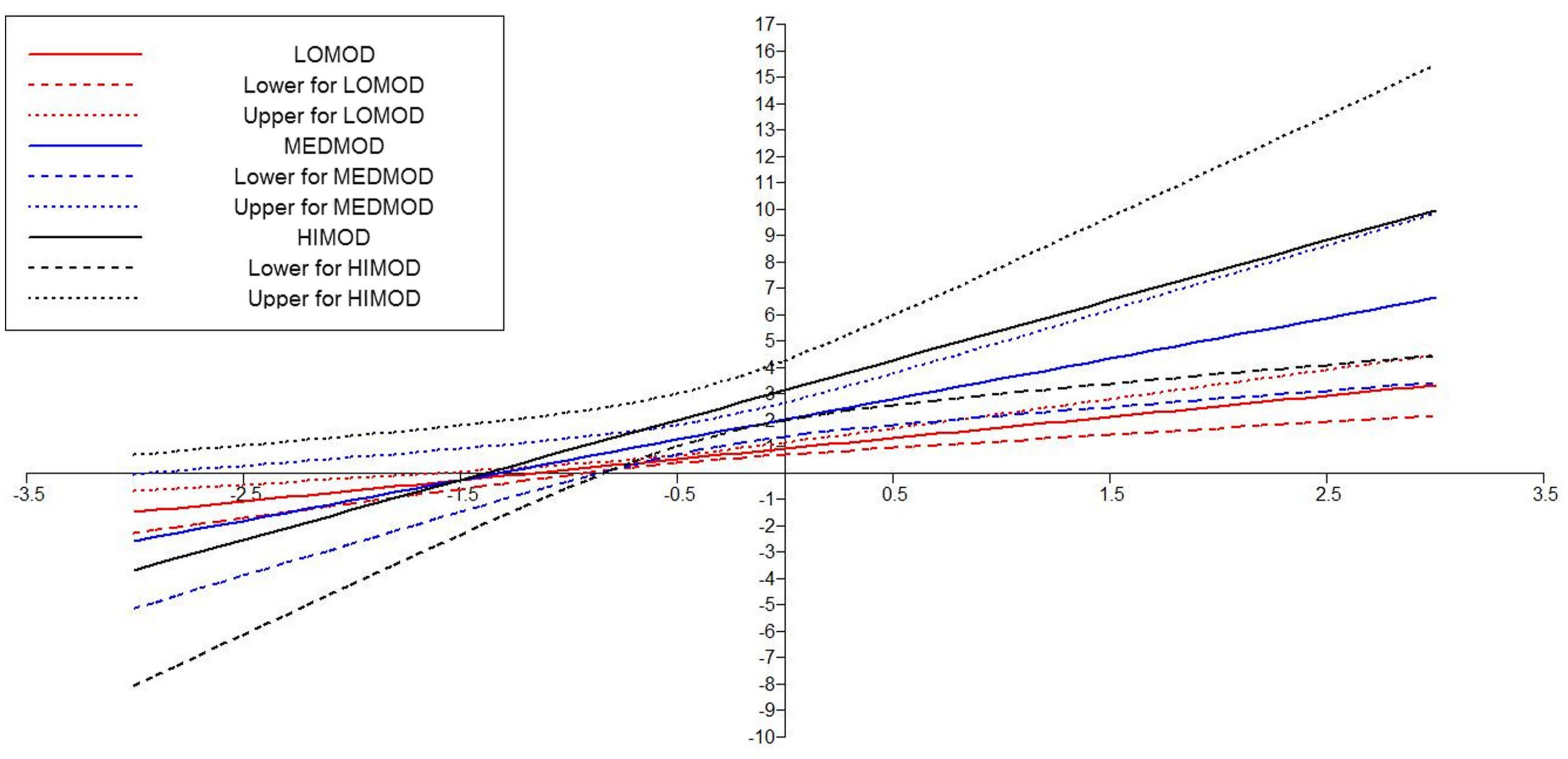
Interaction plot showing the moderating effect of TESOL amplified communication apprehension on informal education—entrepreneurial behavior relationship.

In the Hypotheses 6, it was predicted that CA will positively moderate the relationship between IFED and EB. The results in Table 7 indicated that the direct effect of IFED-EB was *β* = 0.556 (*p* = 0.00) and its 95% CI [0.374–0.739], the direct effect of moderating variable CA-EB was *β* = 0.369 (*p* = 0.00) with 95% CI [0.536–0.855], and the interaction effect of CA X IFED was *β* = 0.245 (0.035) with a 95% CI [0.078–0.431]. Additionally, by conducting Simp-Slope analysis, researchers have further confirmed the moderating effect of CA at different low, medium, and high corresponding values. It was found that higher values of CA moderated the relationship more strongly and positively as compared to medium and lower values. These values indicate that *H*6 was also supported (see Figures 7, 8).

## 5. Implications

Firstly, the theoretical significance of the current research study is to explore the relationship between various elements of entrepreneurship: the relationship between entrepreneurial education, entrepreneurial behavior, and entrepreneurial intention. The study explored the relationships between the above-mentioned variables by integrating TESOL communication apprehension in the framework as a moderator. The results show that formal and informal education has a significant impact on entrepreneurial behavior, and entrepreneurial intention. Entrepreneurial education helps in shaping entrepreneurial behaviors but communication apprehension can significantly reduce the outcomes. High risks and uncertainties are always involved in an entrepreneurial venture, thus communication apprehension increases these risks manifolds. Based on the theory of planned behavior, it is explored in this study that working on TESOL communication apprehension can bring positive outcomes for students and educationists.

Secondly, the moderation model of TESOL communication apprehension, entrepreneurial education, and entrepreneurial behavior has been established by this study. The results depict that TESOL communication apprehension affects the entrepreneurial competence of students.

This paper throw light on the role of formal and informal entrepreneurial education in improving students ‘entrepreneurial intention and behavior of students. This framework improves the theory of the relationship between entrepreneurial education intervariable and also evaluates the impact of communication apprehension on entrepreneurial behavior and intention.

The theoretical implications of this study introduce an advancement in the theoretical framework itself. The study advances and validates the theory of planned behavior. The current study can play a significant role in shaping the entrepreneurial behavior and entrepreneurial intentions of the students. The study highlights the role of TESOL communication apprehension during the entrepreneurial education which can significantly guide students and educationists.

The current study can be used as a practical guideline by psychologists and educationists to learn the role of TESOL communication apprehension and to overcome it during the entrepreneurial learning process. The current study shows that communication apprehension reduces the entrepreneurial intentions of the students. This study has explored that while shaping the entrepreneurial behaviors of the students by using educational strategies, it is important to overcome the fears and anxiety which come with the TESOL communication apprehension. This research gives new insights into the role of TESOL communication apprehension during entrepreneurship education and the entrepreneurial intentions of students. This research and model can be used to assess the impact of entrepreneurial education, communication apprehension, and entrepreneurial intention among the students.

## 6. Conclusion

This research has highlighted and emphasized that the coping policy a person uses to deal with challenging or confounded uncomplimentary and unfavorable situations is crucial since it will have an impact on their psychosocial outcomes, especially their mental health. Positive coping and negative coping are opposites of one another. To meet the objectives of the study, we collected data from the students studying in Chinese universities. A survey questionnaire was designed to collect data from university students who were part of the TESOL program. Data analysis revealed that both FED and IFED significantly predicted EB, while EI was also found to mediate the relationship between formal and informal education and entrepreneurial behavior. Moreover, TESOL amplified communication apprehension also significantly moderated the relationships between formal and informal education with entrepreneurial behavior.

## Data availability statement

The original contributions presented in the study are included in the article/supplementary material, further inquiries can be directed to the corresponding author.

## Ethics statement

All subjects gave their informed consent for inclusion before participating in the study. The study was conducted per the Declaration of School of Foreign Languages, Guangzhou City University of Technology.

## Author contributions

JS did data collection, data acquisition, literature review, and methodology. XH designed the concept and wrote the paper. All authors contributed to the article and approved the submitted version.

## Conflict of interest

The authors declare that the research was conducted in the absence of any commercial or financial relationships that could be construed as a potential conflict of interest.

## Publisher’s note

All claims expressed in this article are solely those of the authors and do not necessarily represent those of their affiliated organizations, or those of the publisher, the editors and the reviewers. Any product that may be evaluated in this article, or claim that may be made by its manufacturer, is not guaranteed or endorsed by the publisher.
